# Changing attitudes about the impact of women's employment on families: The COVID‐19 pandemic effect

**DOI:** 10.1111/gwao.12874

**Published:** 2022-06-30

**Authors:** Leen Vandecasteele, Katya Ivanova, Inge Sieben, Tim Reeskens

**Affiliations:** ^1^ Institute for Social Sciences Swiss Centre of Expertise in Lifecourse Research LIVES University of Lausanne Lausanne Switzerland; ^2^ Department of Sociology Tilburg School of Social and Behavioral Sciences Tilburg University Tilburg The Netherlands

**Keywords:** COVID‐19 pandemic, female employment, gender attitudes

## Abstract

We use representative longitudinal panel data from the Dutch European Values Survey (EVS) to study whether the COVID‐19 pandemic shifted opinions about how a woman's full‐time employment impacts family life. The data was collected before the COVID‐19 pandemic in 2017 and in May 2020. The analysis focuses on groups whose unpaid and paid work situation changed abruptly with the COVID‐19 pandemic: parents with coresident children, and those who experienced a change in paid workload that clashes with traditional gender role expectations, namely women whose workload increased and men whose workload decreased or who stopped working. We found that groups that faced an abrupt change in their paid and unpaid work routines that clashed with their previously held gender attitude changed their gender attitude in alignment with the new paid or unpaid work situation. For women in couple households with children, this meant that they saw a halt in their progression toward gender egalitarian attitudes. For those who experienced a change in paid workload that clashes with traditional gender role norms, it meant stronger progression toward gender egalitarian attitudes. The results are interpreted on the basis of cognitive dissonance theory and exposure theory and placed in the context of previous findings.

## 1 INTRODUCTION

The school and childcare closures precipitated by the COVID‐19 crisis, combined with a general slow‐down in the economy, have raised concerns among policy makers, the public, and academics about the possible socioeconomic repercussions. A chief interest across countries has been the possible gender disparity in the impact of the measures that governments have taken to slow down the spread of the disease (Blaskó et al., 2020). Studies in this area report a behavioral response to the COVID‐19 pandemic in terms of the division of labor within households: though men report to have increased the amount of time they spent on housework and childcare during the pandemic, women still appear to do the biggest share of unpaid work (Carlson et al., 2021; Farré et al., 2021; Shafer et al., 2020; Yerkes et al., 2020). This finding, tied with analyses showing that the negative impact of the crisis on employment has been more marked among women than men, for example, likelihood to drop out of the labor market and decrease in number of hours worked (Adams‐Prassl et al., 2020; Petts et al., 2020), has fueled concerns that the pandemic might constitute a setback to women's position in the workforce, also in the longer run. In order to contribute to the insights in this area, we investigate whether the COVID‐19 pandemic influenced *gender attitudes* about work and family. Specifically, we assess changes to the attitude “family life suffers when the woman has a full‐time job” between a pre‐pandemic measurement and a measurement at the onset of the COVID‐19 pandemic in May 2020. While behavioral adjustments to an ongoing pandemic can shed important light on subsequent (gender) inequalities, our work on gender attitudes^1^ aims to examine people's preferences and ideals that surpass the specific behavioral response and may thus be related to broader changing cultural norms about gender and employment. Therefore, our key contribution is to assess whether an exogenous shock to paid and unpaid work routines, such as caused by the COVID‐19 pandemic, can shift perceptions about how a woman's full‐time employment impacts family life.

Though gender attitudes have sometimes been found to be more egalitarian than behaviors (Cooper, 2000; Kjeldstad & Lappegard, 2014), there is still clear evidence that attitudes are predictive of behaviors and thus, worthy of investigation in their own right (Corrigall & Konrad, 2007; Polavieja, 2015). Importantly for this contribution, a number of studies have shown that gender ideologies can be modified by experiences in individuals' life courses (Baxter et al., 2015; Corrigall & Konrad, 2007; Kroska & Elman, 2009; Schober & Scott, 2012; Zhou, 2017), but scientific work based on a research design with an exogenous shock to life circumstances, such as caused by the COVID‐19 pandemic, is limited, because a truly unexpected change that affects the full population is rare. In the absence of an exogenous shock, it is hard to rule out the influence of unobserved factors that are related to both life transitions such as becoming a parent or increasing the work hours on the one hand and gender attitudes on the other hand. Next to creating a climate of insecurity and stress, the COVID‐19 social distancing measures of school and daycare closures as well as work from home policies and the slowdown of the economy have changed paid and unpaid employment routines abruptly. At the same time, formal and informal care networks often became unavailable. In this contribution, we consider how these unforeseen changes affected individuals' perceptions of the impact of women's employment on families.

A key advantage of our work is that we study individual attitudinal change by using longitudinal representative data from the Netherlands, where the responses to the same questions about individual attitudes were collected prior to and during the ongoing COVID‐19 pandemic. Most studies examining the repercussions of the COVID‐19 pandemic for gendered division of labor have relied on cross‐sectional data where participants were asked to report retrospectively on their pre‐pandemic arrangements next to their current division of labor (Carlson et al., 2021; Farré et al., 2021; Shafer et al., 2020; Yerkes et al., 2020). Such retrospective reports of behaviors and even more so, attitudes, are potentially subject to problems of recall bias (Scott & Alwin, 1998; Tourangeau et al., 2000). The advantage of our data is that we have a reliable prospective measure of our outcome variable, which is a change score between pre‐pandemic gender attitude and the same attitudinal variable in May 2020. The first wave of our data was collected in 2017, whereas the second data collection was carried out between 4 and May 26, 2020 when social distancing regulations and work from home policies were still in place, whereas government‐imposed closures of schools and childcare facilities were in place until 11 May in the Netherlands and only gradually released thereafter. Since our findings are based on a representative panel design and not on cross‐sectional data collection during the crisis only, we are able to more reliably assess actual *changes* in individual attitudes before and during the COVID‐19 pandemic.

## 2 CHANGE IN GENDER ATTITUDES: COHORT REPLACEMENT, LIFE‐COURSE TRANSITIONS, AND EXOGENOUS SHOCKS

The literature presents a clear view with respect to general trends in gender attitudes: Since the 1960s, publics in many countries, including the Netherlands, show increasing support for more egalitarian gender ideologies (see e.g., Inglehart & Norris, 2003; Kraaykamp, 2012). Strictly traditional gender attitudes in line with the male breadwinner model have steeply declined, and there is a steady, albeit slow change in some time periods and/or countries, increase in the support for equal participation of men and women in both the public sphere of work and the private sphere of the family (Knight & Brinton, 2017; Scarborough et al., 2019). This trend toward more progressive gender attitudes has been driven by structural and cultural changes in society, such as increasing levels of education, rise in women's employment, and a decline of religiosity (Thijs, Te Grotenhuis, Scheepers, & van den Brink, 2019). Similarly, backlashes in progress toward gender progressive attitudes, such as those seen in the US context since the 1990s, have also been related to macro‐factors, such as the rise in male overwork (Shu & Meagher, 2018). In fact, much has been written about the finding that despite the major gains for women following their initial mass entry into the labor market, there has been a marked slowdown in the progression toward gender equality in the past few decades, a phenomenon referred to as ‘the stalled revolution’ (England, 2010; Goldscheider et al., 2015; Hochschild, 1989). A focal point of interest in the study of the causes of this ‘stalled revolution’ have been gender attitudes or the endorsement of a division of labor, which is based on separate, gendered spheres of influence (Davis & Greenstein, 2009). In the field of gender attitudes, there is somewhat less evidence of a clear stagnation of a trend toward more equality. Instead, the literature reports a different pace in the trend depending on the type of gender attitude indicator. Though strictly traditional attitudes (on for instance male privilege or a strict male breadwinner model) have declined steeply and continuously, the support for completely equal participation of men and women in both the public sphere of work and the private sphere of family has been less unequivocal (Grunow et al., 2018; Knight & Brinton, 2017; Scarborough et al., 2019). We therefore focus our study of changes in gender attitudes on the perceived trade‐off between women's work and family life with an indicator measuring support for full‐time work for women.

Evidence shows that cohort replacement plays an important role in determining attitudinal change over time, as especially younger birth cohorts have more progressive gender attitudes (Kiley & Vaisey, 2020; Scarborough et al., 2019). Moreover, a number of studies show that gender attitudes change not only with new cohorts but also over the lifetime of individuals with transitions in family and work life (Brewster & Padavic, 2000; Kraaykamp, 2012). A chief focus has been on the transition to parenthood due to the well‐documented mismatch between pre‐birth expressed egalitarian attitudes and the actual post‐birth division of labor (Grunow & Evertsson, 2019). A number of studies have shown that upon the birth of the first child, parents tend to adopt more traditional gender attitudes (Baxter et al., 2015; Corrigall & Konrad, 2007). Interestingly, however, this change in attitudes is contingent on whether the mother remains engaged in the labor market or not, with attitudes of the person becoming more egalitarian in the former case (Kroska & Elman, 2009; Schober & Scott, 2012; Zhou, 2017). This aligns with research on employment transitions: women who work more hours have been shown to become more gender progressive over time (Corrigall & Konrad, 2007). Similarly, Kroska and Elman (2009) show with US panel data that women become more egalitarian following a transition into work and with increased work experience, and their increased work orientation also renders the gender orientation of their husbands more egalitarian, although to a lesser extent. In other words, individuals appear to adjust their attitudes to their lived realities in the sphere of work or family.

In a similar vein, a few studies examined the impact of macro‐level exogenous shocks on gender attitudes, whereby the most important finding is that a sudden downturn in the economic cycle has been related to increased support for gender egalitarian attitudes (Guo & Gilbert, 2012; Távora and Rodríguez‐Modroño, 2018). Again, this might be due to an adjustment of people's attitudes to a changed employment reality in the household. Indeed, recessions typically see a shift away from male breadwinner households and an increase in female breadwinner households (Dotti Sani, 2018), and there is a well‐established added worker effect whereby women take up work as a reaction to the unemployment of their partner (McGinnity, 2002), although this is not observed in all contexts (see work on the Netherlands by Merens & Josten, 2016). A study on workplace location during the COVID‐19 pandemic in the Netherlands found that couples where the male partner worked from home moved more toward gender egalitarian attitudes regarding whether a woman or man is best suited for childcare, in comparison to couples where the man worked away from home (Begall & Verbakel, 2021).

Overall, the number of studies on the impact of exogenous shocks on gender attitudes is rather limited and mainly focus on the impact of an economic shock. While the COVID‐19 pandemic has undeniably been coupled with a downturn in the economic cycle (Eurostat, 2020b), what makes this crisis unique are the clear repercussions of the restriction measures for combining paid and unpaid household labor. This raises the question of the extent to which individual gender attitudes about women's employment might have been impacted by the (perceived) challenges of coordinating the competing needs of domestic and paid labor.

## 3 THEORETICAL FRAMEWORK: GENDER ATTITUDE CHANGE AT THE ONSET OF THE COVID‐19 PANDEMIC

A worldwide pandemic like COVID‐19 is an unprecedented shock in the lives of all human beings. In addition to health worries, the slowdown of the economy and school and daycare closures paired with the unavailability of informal care networks have led to many people experiencing an abrupt change in their usual paid and household work routines. For families, the *unpaid childcare and household work* burden has increased as a result of the unexpected restrictions in outsourcing these activities. In addition, the *paid employment situation* of many individuals changed. While some might have seen their workloads increase, others have seen a reduction in work or have been confronted with (a phase of) unemployment. Many employees have been asked to work from home. In what follows, we develop a theoretical framework to understand how these exogenous shocks could have affected men's and women's perceptions around women's employment and family life. We thereby specifically focus on theories of adaptation to change.

A commonly discussed theory explaining the relationship between work and gender attitudes is the exposure‐based theory. It predicts that individuals adopt egalitarian gender attitudes after exposure to these attitudes through socialization in networks of people with progressive gender attitudes or personal experience (Bolzendahl & Myers, 2004). Change in attitudes are mostly explained over the life course as a result of longer‐term exposure, such as childhood socialization, education, or workplace experience. To give only one example: early childhood socialization in a family with a working mother will lead to more endorsement of progressive gender roles. However, the exposure‐based model has also been used to explain the changed paid and unpaid work situation in couples during the COVID‐19 lockdown (Begall & Verbakel, 2021).

Going more into the psychological adaptation processes, the *cognitive dissonance theory* allows us to understand cognitive adaptations to relatively abrupt changes in circumstances and behaviors. This theory asserts that individuals wish to avoid the feelings of discomfort that stem from cognitive discrepancies between their attitudes, their behavior, and the circumstances they find themselves in (Festinger, 1957). The ways to reduce cognitive dissonance is to either change these attitudes, the behavior or the circumstances, whereby attitudes are often the most easily malleable. Cognitive dissonance theory is often used to explain the fact that individuals tend to adopt more traditional gender views after the birth of their first child in response to having adopted a gendered division of labor to cope with the pressure to combine household and childcare responsibilities (Baxter et al., 2015; Schober & Scott, 2012; Zhou, 2017). The cognitive dissonance theory would suggest that especially groups who, due to the COVID‐19 crisis, have been confronted with an abrupt change in their paid and unpaid work routines that clashes with societal gender norms or their own previously held gender attitude would change their gender attitude to better align with the new paid or unpaid work situation. We will examine the following groups in particular: (1) parents with coresident children and (2) those who experienced a change in paid workload that clashes with traditional gender role expectations, namely women whose workload increased or men whose workload decreased or who stopped working.

Due to childcare and school closures and the unavailability of informal care networks, parents with coresident children especially have experienced an *increased burden of unpaid work* that may cognitively be hard to reconcile with a progressive gender role attitude. Indeed, while traditional gender norms imply a strict division of labor whereby men engage in paid and women in unpaid employment, the provision of public childcare has been shown to lead to more gender egalitarian work patterns (Zoch & Hondralis, 2017) and is related to progressive gender attitudes (Grunow, 2019). Hence, if the adoption of more equal gender roles is facilitated by the availability of childcare options, the sudden unforeseen restriction in the ability to outsource childcare, schooling, and unpaid labor creates a new reality of work–family conflict that may lead to cognitive dissonance with progressive gender role preferences concerning women's engagement in full‐time employment. The cognitive dissonance in this situation is expected to be larger for women because of prevailing cultural expectations that women take on the majority of these unpaid household and care tasks. The trend toward gender equality in attitudes and female employment has coincided with a slow pace of change of men's participation in unpaid work at home, with employed women typically engaging in housework and caregiving in the “second shift” after her paid job finishes (Hochschild, 1989; Hook, 2010). In previous research, transition to parenthood and its associated unpaid work surge has been shown to move both men and women toward more traditional gender attitudes (Baxter et al., 2015; Corrigall & Konrad, 2007). However, we expect that mothers might experience a stronger effect given the cultural expectation to take on the larger part of unpaid work at home. We thus expect that *parents with children shift more toward gender traditional attitudes than individuals in other household types (H1) and this effect is stronger for mothers than for fathers (H2).*

In addition, the *paid employment situation* of many individuals changed. The workload of some workers, for example, those in so‐called essential professions among which many women (Yerkes et al., 2020), might have increased, while others have seen reduction in work or have been confronted with (a phase of) unemployment (Yerkes et al., 2020). Kroska and Elman (2009) demonstrated that employment changes shift gender attitudes, especially if the employment change entails a shift away from gendered patterns; for example, women who move back into employment or who increase their work experience or occupational status have been shown to move toward more egalitarian gender attitudes. Combining exposure and cognitive dissonance theory, it is expected that exposure to this new employment situations creates cognitive dissonance between a more traditional gender attitude and a less traditional employment situation and as a result, individuals may shift their gender attitude to align with their new employment situation. We expect that individuals who experienced a change in employment situation that makes their new employment situation clash with traditional gender role expectations will adopt a more progressive gender attitude. This is the case for people who move from more gender‐typical to more gender‐atypical work situations, like women who see their paid workload increase or men who see their paid workload decrease. Note that not only clear shifts in employment status, but also individuals' assessments of changes in their workload might create discomfort stemming from cognitive dissonance between the perceived workload and someone's gender attitude. We thus expect that w*omen whose perceived paid workload increased during the COVID‐19 pandemic shift more strongly toward a progressive gender attitude than women whose perceived paid workload did not change (H3) and men whose perceived paid workload decreased during the COVID‐19 pandemic or who became (temporary) unemployed shift more strongly toward a progressive gender attitude than men whose workload did not change (H4).*

## 4 THE DUTCH CONTEXT

Though the theoretical mechanisms discussed point to potentially universal effects of the pandemic on gender attitudes, our study uses data from one particular national context, the Netherlands. It therefore is important to note the COVID‐19 related measures implemented by the Dutch government prior to our second wave of data collection. In the Netherlands, COVID‐19‐related school and daycare closures began in mid‐March 2020, with almost complete closures lasting until May 11, 2020 (Hale et al., 2020). Once reopened, schools and childcare facilities had to observe strict social distancing measures resulting in children being able to attend for about half the usual time. The social distancing measures also led to the unavailability of paid household help and informal care networks (such as grandparents), which are widely used in the Netherlands (Roeters et al., 2016). There was no provision of paid corona‐related parental leave. Regular parental leave options, which are below the OECD average in any case (OECD, 2021), cannot be used in acute situations, while emergency leave typically is limited to a few days only. Moreover, the government imposed closing of or working from home for all‐but‐essential workplaces (Hale et al., 2020). All these measures put substantially more strain on families in combining work and family life.

It should be mentioned that labor market participation of women in the Netherlands is rather unique compared to other European countries (Brakel & Merens, 2018). Employment rates among Dutch women have been consistently high over the past decade, with 74.1% employed in 2019 (Eurostat, 2020a). Yet, only a minority of these women are employed full‐time: 24.8% in 2019 (Eurostat, 2020c). This pattern of high part‐time employment among women has been pointed out as a key reason why the proportion of female managers in the Netherlands is one of the lowest in the EU (Brakel & Merens, 2018) and why about 40% of Dutch women are not financially independent (Herbers & Portegijs, 2018). There is a strong cultural preference for part‐time work in order for families to be able to take care of their children, which is reinforced by institutional arrangements (Knijn, 1994; Yerkes & Visser, 2005). Formal childcare services are typically not used full‐time and many families rely on informal care networks like grandparents for childcare (Roeters et al., 2016).

## 5 MATERIALS AND METHODS

### 5.1 Data

We analyze Dutch data from the European Values Study (EVS, 2020; see www.europeanvaluesstudy.eu), supplemented with time series on longer‐term trends from the Dutch samples of the International Social Survey Program (ISSP; see http://w.issp.org/menu‐top/home/). New data was collected in May 2020, when the online respondents to the 2017 Dutch sample of the European Values Study were reapproached with the same questions they answered in 2017. The 2017 data was collected through a mixed mode strategy, combining face‐to‐face data collection with an online survey integrated in the LISS Panel (Longitudinal Internet Study for the Social Sciences), a probability‐based household panel. For the 2017 data collection—which ran between September 1, 2017 and 31 January 2018—approximately 2500 respondents were invited to participate, resulting in 2053 participating respondents. The 2020 data collection consisted of reapproaching the 2017 LISS Panel online respondents between 4 and May 26, 2020 and resulted in a sample of 1288 respondents who participated in both the 2017 and 2020 wave. The attrition between the 2017 and 2020 data collection amounts to 38% and is related to gender, age, and household type, but not to educational level and our attitudinal variable of interest “Family life suffers when a woman has a full‐time job”. Appendix A provides more information on the patterns of attrition for our subsample. The analyses were restricted to respondents of labor active age at both time points, that is, the respondents were aged 18–64 in the 2017 wave and between age 20 and 66 in the 2020 follow‐up wave. Note that the official retirement age in the Netherlands was 66 years and 4 months in 2020. Furthermore, respondents with item nonresponse on the variables of interest were excluded, resulting in an analytical sample of 675 respondents (314 men and 361 women).

### 5.2 Variables

**FIGURE B1:**
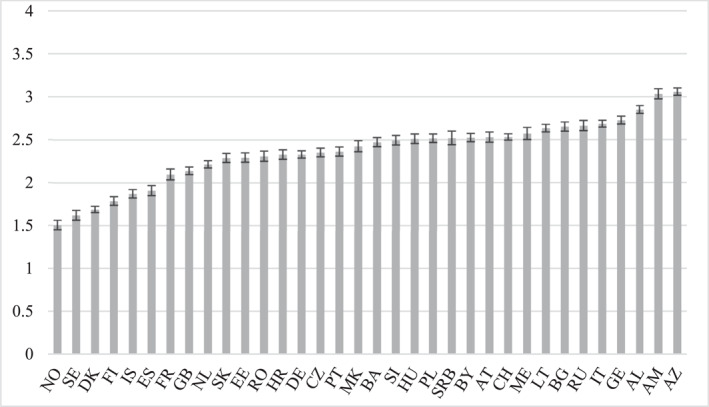
Agreement with statement “Family life suffers when the woman has a full‐time job”. European Values Studies, 2017. Analytical sample: age 18‐67. Weighted data Agreement with statement. 1 Strongly disagree; 2 Disagree; 3 Agree; 4 Strongly agree

The gender attitude variable of interest taps into gender traditionalism and measures the level of agreement with the following statement: “Family life suffers when the woman has a full‐time job”. We reverse‐coded the response categories from the EVS so that they vary in our analyses between (1) “strongly disagree” and (4) “strongly agree”. This attitude item is particularly suitable for the Dutch context where employment rates among women are particularly high, but only a quarter of working women are employed full‐time (Eurostat, 2020c). However, this does not mean that the attitudes toward full‐time female employment are uniform across the Dutch population. It is notable that among the battery of five gender role attitudes in the 2017 round of the European Values Studies, the item Family life suffers when the woman has a full‐time job has a larger level of variation in the Dutch population than the other gender role items (Table B1. In Appendix B). Given that this was the only gender attitude item that was repeated in the 2020 data collection, we used Dutch LISS data from 2018 to 2019 to verify the correlation between this item and other gender role attitude items. Tables B2 and B3 demonstrate that the item correlates strongly with other gender attitude items on working mothers and the division of labur. Therefore, despite it being the only item available for our analysis, we were confident that it is, at least in the Dutch context, a good representation of gender attitudes toward working women. Moreover, in the European perspective, the Netherlands shows rather similar values on this variable as countries like France, Great Britain, Slovakia, or Germany on our variable of interest. It is thereby situated among a large group of countries in the middle part of the distribution as seen in Figure B1 in Appendix B. The analysis is based on the difference in score between 2020 and 2017, whereby higher values indicate a move toward more gender traditionalism and increased agreement with the statement “family life suffers when the woman has a full‐time job” in 2020 compared to 2017.

The household type variable distinguishes between single and couple households (including married and unmarried couples) and whether children were present in the household. Adult respondents living with their parents were excluded from the analysis in order not to conflate parents and children in the originally provided household type “couple with children”. For couple households without children, we make a distinction between respondents who are potentially pre‐children aged <40 and those aged ≥40. The reason is that couples who are of potentially child‐bearing age might be more alert to the changes that the COVID‐19 pandemic entails for the difficulty of combining work life with a family.

The 2020 questionnaire included two questions related to the work situation respondents experienced during the COVID‐19 pandemic. The first question inquired about changes in the workplace since the outbreak of the COVID‐19 pandemic, with response categories (1) ‘I work more often from home’, (2) ‘I work at my usual workplace’, (3) ‘I am temporary unemployed or laid off’, and (4) ‘I had no paid job before the pandemic and at this moment I still don't have one’ (reference category). The second question asked about the workload of the respondent, measuring whether participants have seen their workload increase, decrease, or remain the same during the COVID‐19 pandemic.

Next to these variables of interest, we controlled for age, highest education level (Low, primary school or vmbo; Middle, havo/vwo or mbo; and High, hbo or wo), and whether there has been a change in the presence of children in the household between the two moments of data collection in 2017 and 2020. Furthermore, all analyses are controlled for whether the interview took place before or after 11 May, the date when the strictest measures were gradually being released, and school and childcare facilities gradually reopened. Alternative models controlling for the date of interview itself are not presented but provided similar results. Descriptive statistics of all variables in the analyses can be found in Table 1. It must be noted that the sample size in some of the subgroups is small, which limits the scope of the analysis, notably also in terms of adding interaction terms.

**TABLE 1:** Descriptive statistics

Variable	Men (*N* = 314)				Women (*N* = 361)			
	Continuous variables		Categorical variables		Continuous variables		Categorical variables	
	Scale range	Mean (SD)	N	%	Scale range	Mean (SD)	N	%
Dependent variable								
Difference score 2020–2017 family life suffers when the woman has a FT job	−3 to 3	−0.213 (0.801)			−2 to 2	−0.241 (0.703)		
Independent variables								
Family life suffers when woman has a FT job (2017)	1–4	2.127 (0.836)			1–4	2.177 (0.926)		
Household‐type								
Single			100	31.85			100	27.70
Couple without children, age <40			16	5.10			27	7.48
Couple without children, age >=40			90	28.66			87	24.10
Couple with children			88	28.03			114	31.58
Other			20	6.37			33	9.14
Corona crisis work situation								
Works more often from home			128	40.76			118	32.69
Works at usual workplace			113	35.99			126	34.90
Temporarily unemployed or laid off			11	3.50			20	5.54
No paid employment since before corona crisis			62	19.75			97	26.87
Corona crisis workload								
More work than before corona crisis			36	11.46			60	16.62
About as much as before			156	49.68			135	37.40
Less work than before corona crisis			49	15.61			49	13.57
Temporarily unemployed or laid off			11	3.50			20	5.54
No paid employment since before corona crisis			62	19.75			97	26.87
Age in 2020	20–66	50.53 (11.94)			22–66	48.33 (12.5)		
Education level								
Low			58	18.47			72	19.94
Middle			98	31.21			125	34.63
High			158	50.32			164	45.43
Change of presence of child(ren) in household								
No change			271	86.31			319	88.37
None 2017, child(ren) 2020			14	4.46			17	4.71
Child(ren) 2017, none 2020			29	9.24			25	6.93
Interview timing								
4−May 10, 2020			198	63.06			239	66.20
11–26 May 2020			116	36.94			122	33.80

*Note*: Data: European Values Study, the Netherlands 2017, 2020. Unweighted data. Dependent variable: Change score 2020–2017 of agreement with the statement “Family life suffers when woman has a full‐time job”. Higher values on the change score indicate a move towards gender traditionalism, lower values indicate a move away from gender traditionalism.

### 5.3 Statistical model

The main statistical model consists of a conditional change score model (Allison, 1990; Taris, 2000). Because of our interest in individual change in the gender attitude between 2017 and 2020, the dependent variable is measured as a change score whereby the 2017 response is subtracted from the 2020 response. To control for the occurrence of regression to the mean due to floor and ceiling effects, the gender attitude variable in 2017 is introduced as a control variable. This makes the model equivalent to the regressor variable method (Werts & Linn, 1970, cited in; Allison, 1990). Ordinary least squares regression techniques are used and post‐stratification weights are applied to all analyses to correct for the Dutch population distribution with regard to sex, age, education, and region. The point estimates of the unweighted analyses only slightly changed compared to the weighted analyses, and the overall pattern of findings was the same.

## 6 RESULTS

Before examining changes in gender attitudes after the onset of the COVID‐19 pandemic, we present a longer time trend of the specific gender attitude studied here for the Netherlands. This will allow us to interpret current changes against the backdrop of the existing time trend. Subsequently, we employ the longitudinal change score model to examine the change that occurred between the pre‐measurement and the COVID‐19 data collection.

**FIGURE 1:**
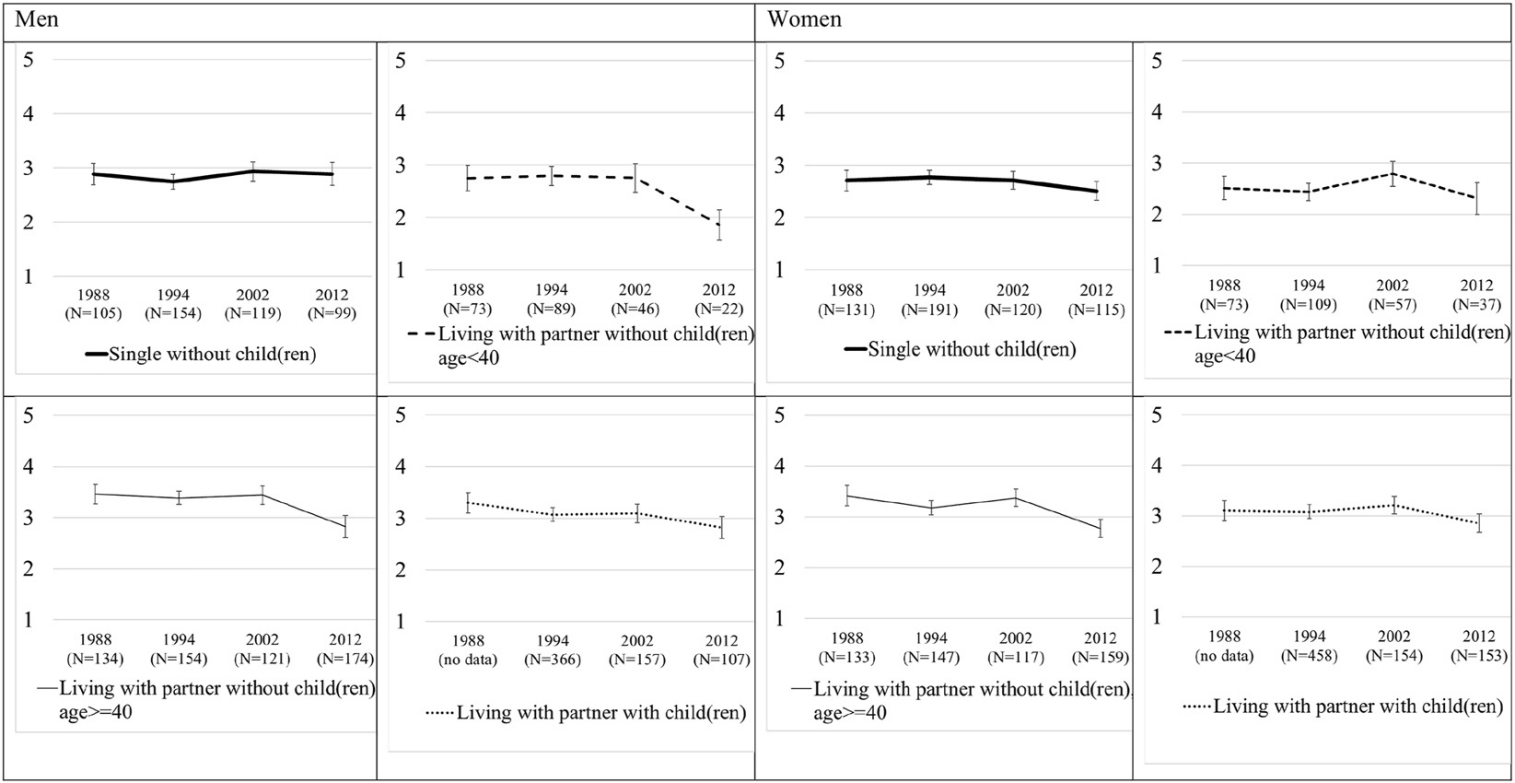
Agreement with statement “Family life suffers when the woman has a full‐time job”, by household type. *Source*: Data: International Social Survey Program, the Netherlands. Analytical sample age 16–70. Weighted data. Average agreement with statement “Family life suffers when woman has a full‐time job”: 1, Strongly disagree; 2, Disagree; 3, Neither agree nor disagree; 4, Agree; and 5, Strongly agree. 90% confidence intervals. Note that the single with children group is not presented due to small case numbers

In Figure 1, we present time trend data from repeated cross‐sections of the International Social Survey Program for the Netherlands which, unlike other surveys, includes a longer‐time trend on the gender item of interest. The average level of agreement with the statement “Family life suffers when the woman has a full‐time job” is presented from 1988 until 2012, broken down by gender and different household types. In general, the Dutch population neither agrees nor disagrees with the statement that family life suffers when the woman has a full‐time job. To‐ward the end of the observed time series, there is a development toward more progressive views on women's full‐time employment.

As explained in the theoretical section, attitudinal change often happens through cohort replacement but it has to be noted that the descriptive nature of Figure 1 does not allow us to disentangle age, period, or cohort effects. For both men and women, singles without children and those living with a partner without children under age 40 have the most progressive gender attitudes at the beginning of the time series in 1988 (lowest agreement with the statement), whereas those living with a partner and children have less progressive views as well as childless couples over age 40. Over time, all groups become more progressive, especially toward the end of the time series, with one exception: the attitudes of single males remain more or less constant. Although there is a time gap of five years between the latest ISSP round and our longitudinal EVS data, we tentatively conclude that there is a trend toward more progressive gender norms in the Netherlands before the COVID‐19 pandemic.

**FIGURE 2:**
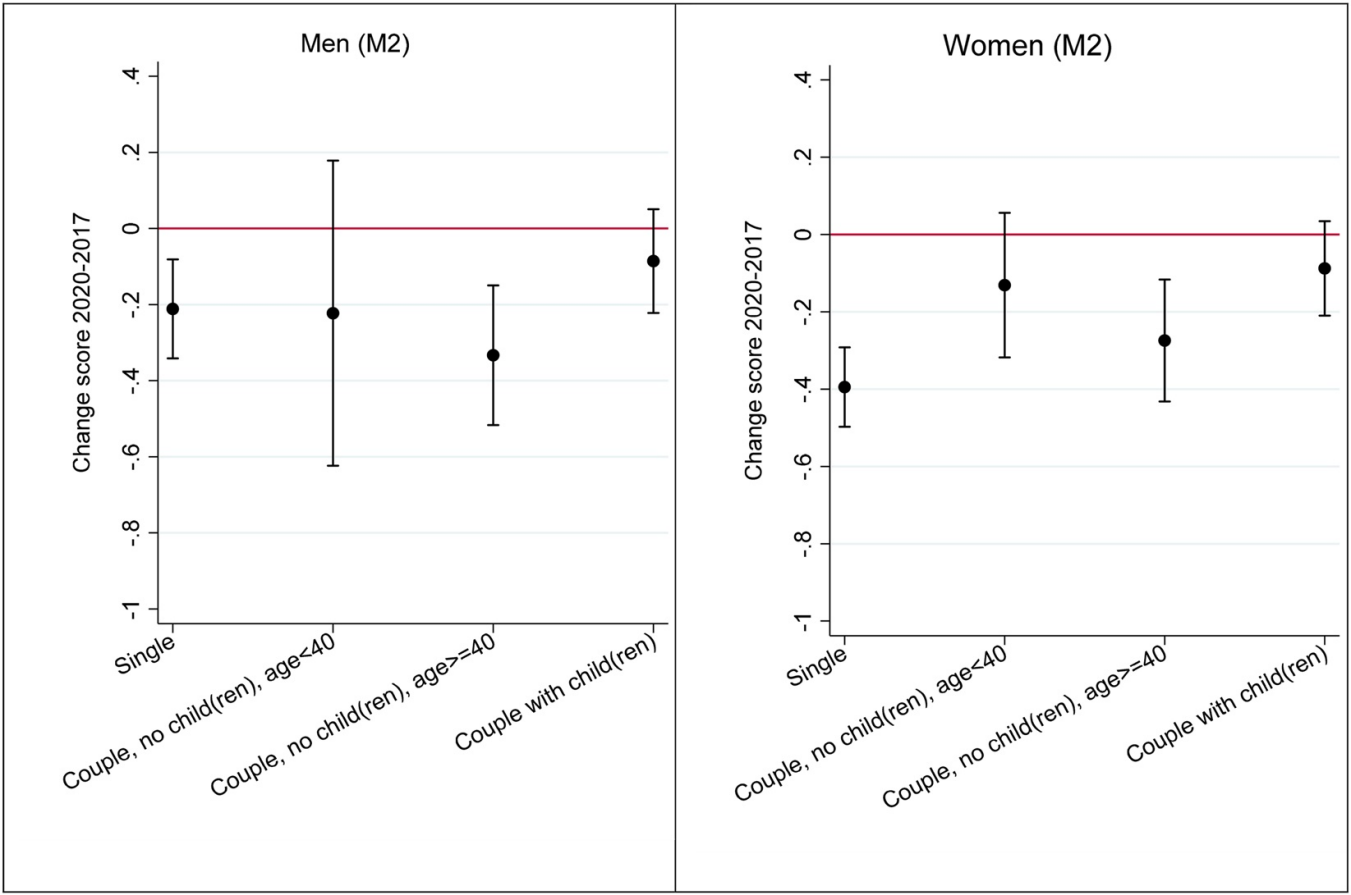
Predicted values of change score by household type. *Source*: Data: European Values Study, the Netherlands, 2020 and 2017. Change score 2020–2017 of agreement with the statement “Family life suffers when woman has a full‐time job”. Higher values on the change score indicate a move toward gender traditionalism, lower values indicate a move away from gender traditionalism. 90% confidence intervals. Weighted data

**FIGURE 3:**
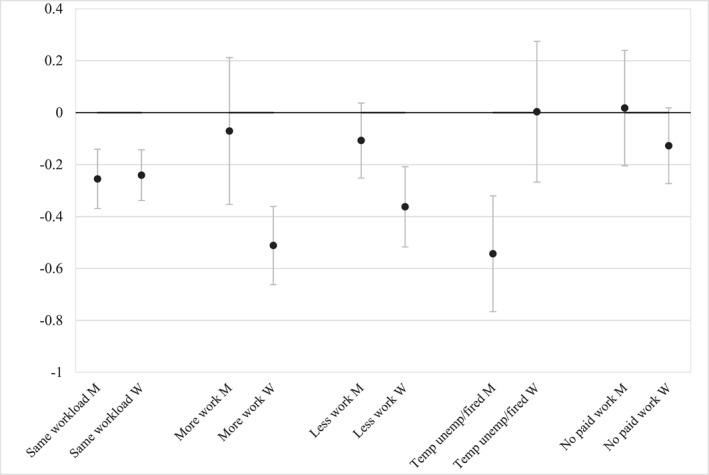
Predicted values of change score by corona crisis workload variable. *Source*: Data: European Values Study, the Netherlands, 2020 and 2017. Predicted values on the basis of M2 Men and M2 Women. Change score 2020–2017 of agreement with the statement “Family life suffers when woman has a full‐time job”. Higher values on the change score indicate a move toward gender traditionalism, lower values indicate a move away from gender traditionalism. 90% confidence intervals. Weighted data

In Table 2, the results of the conditional change‐score models are presented. We present two models per gender group: one with the COVID‐19 work situation variable and one with the COVID‐19 workload variable as these variables could not be presented in the same model due to overlap in the category of “no paid employment since before the COVID‐19 pandemic”. The predictor variables of interest are household type and the COVID‐19 pandemic variables, and the models are controlled for age, education level, and whether there was a change in the presence of children in the household between 2017 and 2020. Models without control variables showed slightly smaller effect sizes but, overall, very similar results. Figures 2 and 3 display attitudinal change scores based on the models, which makes it easier to interpret the results. Here, a positive predicted value on the change‐score indicates more agreement in 2020 than in 2017 with the statement “Family life suffers when the woman works full‐time”, whereby a change of 1 equates a move between the categories (e.g., from “agree” to “strongly agree” or from “disagree” to “agree”). Thus, whereas a positive change‐score indicates a move toward gender traditionalism, a negative change‐score indicates a move toward more progressive gender attitudes. The zero line indicates no change in the gender attitude. However, it should be noted that our hypotheses were based on comparisons between groups rather than on whether a specific group did or did not experience change in attitudes. Therefore, it is important to pay attention to the potential overlap in the confidence intervals between the specific groups and the reference group.

**TABLE 2:** Conditional change‐score regression analysis “Family life suffers when the woman works full‐time”, change scores 2020–2017

	M1 Men	M2 Men	M1 Women	M2 Women
Family life suffers when woman has FT job (2017)	−0.470*** [−0.575; −0.365]	−0.477*** [−0.583; −0.372]	−0.381*** [−0.454; −0.308]	−0.377*** [−0.449; −0.304]
Household‐type (ref. Single)				
Couple without children, age < 40	−0.021 [−0.440; 0.397]	−0.011 [−0.438; 0.416]	0.254^+^ [0.036; 0.472]	0.264* [0.048; 0.479]
Couple without children, age ≥ 40	−0.102 [−0.317; 0.114]	−0.122 [−0.332; 0.088]	0.093 [−0.096; 0.282]	0.120 [−0.066; 0.307]
Couple with children	0.146 [−0.060; 0.352]	0.126 [−0.079; 0.330]	0.299** [0.138; 0.461]	0.307** [0.146; 0.469]
Other	0.181 [−0.202; 0.563]	0.145 [−0.247; 0.537]	−0.039 [−0.253; 0.174]	−0.030 [−0.241; 0.181]
Corona crisis work situation (ref. Works at usual workplace)				
Works more often from home	−0.086 [−0.292; 0.119]		−0.050 [−0.210; 0.110]	
Temporarily unemployed or laid off	−0.380* [−0.660; −0.099]		0.321^+^ [0.036; 0.606]	
No paid employment since before Corona crisis	0.192 [−0.054; 0.437]		0.181 [−0.002; 0.364]	
Corona crisis workload (ref. As much as before)				
More work than before Corona crisis		0.184 [−0.120; 0.489]		−0.271* [−0.447; −0.094]
Less work than before Corona crisis		0.148 [−0.029; 0.324]		−0.122 [−0.303; 0.059]
Temporarily unemployed or Laid off		−0.288^+^ [−0.557; −0.020]		0.244 [−0.043; 0.532]
No paid employment since before corona crisis		0.273^+^ [0.020; 0.526]		0.113 [−0.067; 0.293]
Age	−0.013* [−0.022; −0.004]	−0.012* [−0.020; −0.004]	0.006 [−0.001; 0.013]	0.005 [−0.002; 0.012]
Education (ref. Low)				
Middle	−0.126 [−0.376; 0.124]	−0.116 [−0.371; 0.139]	0.040 [−0.144; 0.224]	0.027 [−0.159; 0.213]
High	−0.155 [−0.437; 0.127]	−0.184 [−0.435; 0.066]	−0.019 [−0.203; 0.166]	−0.026 [−0.204; 0.151]
Change of presence of child(ren) in household (ref. No change)				
No child(ren) 2017, child(ren) 2020	0.137 [−0.222; 0.497]	0.187 [−0.182; 0.556]	−0.012 [−0.359; 0.336]	−0.017 [−0.350; 0.315]
Child(ren) 2017, no child(ren) 2020	0.110 [−0.178; 0.397]	0.118 [−0.162; 0.399]	0.029 [−0.198; 0.255]	0.000 [−0.225; 0.225]
Interview before May 11, 2020	−0.145 [−0.306; 0.015]	−0.140 [−0.300; 0.020]	−0.087 [−0.232; 0.057]	−0.108 [−0.254; 0.039]
Constant	1.582*** [0.963; 2.201]	1.472*** [0.873; 2.070]	0.164 [−0.224; 0.551]	0.276 [−0.130; 0.682]
Observations	314	314	361	361
*R* ^2^	0.299	0.304	0.257	0.273

*Note*: Data: European Values Study, the Netherlands 2020 and 2017. Weighted data. Dependent variable: Change score 2020–2017 of agreement with the statement “Family life suffers when woman has a full‐time job”. Higher values on the change score indicate a move toward gender traditionalism, lower values indicate a move away from gender traditionalism. 90% confidence intervals in brackets.

^+^p < 0.1, *p < 0.05, **p < 0.01, ***p < 0.001.

Our first hypothesis stated that parents, and especially mothers, with children would shift more toward traditional gender attitudes compared to other household types. First, from Figure 2 it becomes clear that none of the groups experience a backlash toward more traditional gender attitudes. The reference group of singles moves most clearly toward a progressive gender attitude. The Models in Table 1 show differences according to household type in gender attitude change, but only for women. Women in couple households with children, and to a lesser extent women aged <40 in couple households without children, report significantly less progression toward endorsing progressive attitudes than the reference category of single households. However, for men, there are no significant differences between the household types in gender attitude change between the two measurement points. This is also shown in Figure 2, in which the predicted values on the change‐score based on M2 Men and M2 Women are presented by household type. The predicted values for men show no significant differences between the household types. The graph for women shows that whereas single women have made a move of 0.4 toward less agreement with the statement and therefore toward a more progressive gender attitude, this is not the case for women in couple households with children and young women in couple households without children. For both these groups, the confidence intervals of the predicted values include the zero‐point of no change. We thus find partial support for hypothesis 1 and 2 which stated that people in couple households with child(ren) and especially women in couple households with child(ren) shift toward traditional gender attitudes. While we do find that women with partner and child(ren) have a less strong shift toward gender progressive attitudes than singles, this effect is absent for men in couple households with child(ren). The finding that young women in couple households without children do not change their gender attitudes is also interesting as we observed a long‐term trend toward more progressive gender attitudes in this group (see the results with ISSP data displayed in Figure 1). The EVS data show that in 2020, this trend has continued mainly for single women and women aged 40+ in couple households without children, but not for mothers with children and women of childbearing age in couple households without children. The result for women of childbearing age in couple households without children is interesting since this group has not been confronted with the repercussions on unpaid work of the school and daycare closures to the same extent as women with children. However, in line with exposure‐based theory (Bolzendahl & Myers, 2004), it is conceivable that exposure through media reports and personal networks of the difficulties experienced by working parents may have had an impact on these women of childbearing age.

Changes in the place of work do not seem to have much impact on gender attitudes, as there are no significant differences between those working from home and those working at the usual workplace (M1 Men and M2 Women, Table 2). In Model 1, we furthermore see that for men being temporarily unemployed or laid off is associated with a negative coefficient and thus a stronger change toward less agreement with the statement indicating more change toward progressive gender attitudes than for the reference group, whereas for women the effect goes in the opposite direction. These results are also displayed in Figure 3.

The expectation in hypotheses 3 and 4 was that individuals whose workload changed toward a gender‐atypical pattern would shift more toward progressive gender attitudes than individuals without change in their perceived workload. We specifically focused on transitions to a gender‐atypical workload, that is, women whose paid workload increased (H3) and men whose paid workload decreased or who became (temporarily) unemployed (H4) during the COVID‐19 pandemic. The hypotheses thus predict different effects for men and women. Model 2 does indeed show different effects for men and women for the categories ‘workload increase’ and ‘(temporarily) unemployment’, but our hypothesis is not confirmed for ‘workload decreases’. The negative coefficient in Table 2 shows that women with more workload than before the COVID‐19 pandemic moved more in the direction of disagreement with the statement that family life suffers when a woman works full‐time than the reference group with no workload change. Also in Figure 3, we see that this group moved about half a category in the direction of a progressive gender attitude, which is double that of women whose paid workload remained constant. The predicted values in Figure 3 further show that the same effect cannot be found for men: for men, more work is associated with no significant change in the gender attitude variable. According to our theoretical frame, women who are exposed to a higher workload move in a gender‐atypical direction, and therefore experience cognitive dissonance between prevailing gender norms and their personal shift in workload. As a result, the gender attitude of these women moves in a gender progressive direction. The same was not expected for men who were exposed to an increased workload because that largely corresponds to prevailing gender norms. Hence, our results lead us to conclude that hypothesis three is confirmed.

On the basis of the same theoretical frames, we expected that men whose perceived paid workload decreased during the COVID‐19 pandemic or who became (temporarily) unemployed would shift more strongly toward a progressive gender attitude than men whose workload did not change (H4). Table 2 shows that men who became (temporarily) unemployed shift more toward a progressive gender role orientation than men without changes in their workload, which provides support for H4. Indeed, theoretically we expected that losing one's job for men is at odds with gendered expectations around the male breadwinner. Therefore, men who are exposed to this situation might experience cognitive dissonance between a gender‐typical attitude and a gender‐atypical work situation and therefore shift their attitudes to a less gender traditional direction. However, the same pattern cannot be found for men who worked less during the COVID‐19 pandemic as they do not differ significantly from men without changes in their workload. Figure 3 confirms this partial evidence for Hypothesis 4. For men, being temporarily unemployed or laid off is associated with a move of almost 0.55 toward more agreement with full‐time work for women, which again is a change that is double in size than that for men who keep the same workload. For women, on the other hand, being (temporarily) unemployed results in no movement toward more progressive gender roles (confidence intervals overlap with zero‐line of no change). However, for men who worked less since the COVID‐19 pandemic, we do not see the same pattern, as this group has no significant change in their gender attitude.

## 7 CONCLUSION AND DISCUSSION

This study set out to examine whether the COVID‐19 pandemic influenced gender role attitudes on work and family by examining the Dutch population's viewpoint on the effect of women's full‐time employment on family life. By focusing on the exogenous shock of the pandemic, which fundamentally impacted individuals' experiences with paid and unpaid labor, our work builds on previous contributions which have examined how gender ideologies can be modified by experiences in individuals' life‐courses. Our findings, based on Dutch longitudinal data from the European Values Study and analyses of within person changes, indicate that no clear backlash toward women's full‐time employment can be detected following the outbreak of the pandemic. In general, the trend toward more progressive gender norms in the Netherlands that we observed in the ISSP data, seems to be prolonged. However, we observed heterogeneity in the impact of the crisis, whereas the progression toward higher acceptance of women's full‐time employment was halted for some groups (notably, for women living with a partner and children); this same progression toward more gender progressive norms was clearly more pronounced for individuals whose experience with (un)paid work during the outbreak of the pandemic was less congruent with a gender‐traditional division of labor (notably, women who reported a higher workload during the COVID‐19 pandemic and men who lost their jobs). We elaborate on these findings further below.

A frequent observation in previous empirical works on life‐course transitions and individual gender ideologies is the fact that people not only select into different life‐course trajectories based on norms and values but that they also actively adjust their ideologies based on their lived experiences (Baxter et al., 2015; Corrigall & Konrad, 2007; Corrigall & Konrad, 2007). This finding has been interpreted in light of the cognitive dissonance theory's (Festinger, 1957) proposition that people wish to avoid the feelings of discomfort that stem from cognitive discrepancies between their attitudes, their behavior, and the circumstances they find themselves in. We contend that our findings can also be understood in light of that theoretical proposition, namely that groups who were exposed to a change in unpaid or paid workload at the onset of the COVID‐19 crisis, adjusted their gender attitude to be more congruent with the new paid or unpaid work routine.

Due to the daycare and school closures as well as the restriction in outsourcing housework, parents and especially mothers with coresident children experienced a substantial increase in unpaid workload (Yerkes et al., 2020). The ensuing work–family balance conflict may make it cognitively hard to maintain strong support for the viewpoint that family life does not suffer when women work full‐time. Similarly, individuals who were exposed to a change in paid workload that clashes with traditional gender role expectations, namely women whose workload increased and men who stopped working, move toward stronger endorsement of progressive attitudes than those who did not experience a change in workload. For women, having more work to do and at the same time disapproving of women working full‐time would lead to cognitive dissonance, which could be a reason why these women move toward more progressive gender attitudes in a time where their workload increased. For men, the results could be explained by the same mechanism. For them, being (temporarily) unemployed entails a move away from the traditional gender role model of being the main breadwinner. This could trigger a move toward more progressive gender attitudes.

What is important to reflect on here is that we did not find evidence of backlash against women's full‐time employment (i.e., a move toward *more* disapproval) for any of the examined groups. However, we do not see this as indicative of lack of effect of the crisis. For example, we found that those who were less likely to have faced a fundamental shift in the need to combine paid and unpaid labor (e.g., singles) had moved toward more progressive attitudes. This is in line with the general trend toward more progressive gender attitudes (Kiley & Vaisey, 2020; Scarborough et al., 2019). What we find noteworthy, however, is that for some groups, that progression might be stalled, for example, mothers with a partner and children. This finding can potentially be linked to the ‘stalled revolution’ in gender equality on the labor market (England, 2010; Goldscheider et al., 2015; Hochschild, 1989). If indeed, individuals' move toward *more* progressive attitudes is halted as they face challenges in dividing their paid and unpaid labor, then we are probably rather unlikely to see further changes in actual behaviors. What remains an open question in our work is whether what we are observing is a temporary effect or whether the impact on individual attitudes will be a long‐lasting effect. If individuals continuously adjust their attitudes to their lived realities, and these work situations quickly change back, we might in fact be observing only temporary changes. However, if gender‐atypical work patterns have shifted more durably (e.g., women increasing their paid workload), this might provide the conditions for longer lasting gender norm changes. Similarly, given that the initial school closures of Spring 2020 were followed by an even longer school closure period in the subsequent fall and winter in the Netherlands, it is possible that what we observed is indicative of pronounced, longer lasting shifts in different groups' acceptance of women's full‐time employment.

An important advantage of our work was the fact that we were able to test our hypotheses using representative longitudinal panel data from the Dutch *European Values Studies* sample that was reapproached at the onset of the COVID‐19 pandemic. In comparison to previous studies that examined attitude change in relation to employment shifts, this study has the advantage that the changes in unpaid work burden and the paid work situation can be treated as exogenous, as they were the direct result of the COVID‐19 pandemic and its associated health policy measures. Whereas there are a few previous studies that have also relied on longitudinal follow‐up (Corrigall & Konrad, 2007; Kroska & Elman, 2009; Schober & Scott, 2012; Zhou, 2017), in the absence of an exogenous shock it is hard to rule out the influence of unobserved factors that are related to both the employment situation and the gender attitude.

At the same time, our findings should be viewed in light of several important limitations. Foremost, we analyzed only one gender attitude item (“family life suffers when a woman works full‐time”), while our theoretical arguments and discussions do at times refer to more general indices of gender‐traditional/egalitarian attitudes. Whereas this decision was driven by the fact that this was the only item available in the 2020 data collection, we also believe that the item is highly appropriate to capture perceptions of women's employment, given the Dutch context where a very high proportion of women are employed but mostly only part‐time (Brakel & Merens, 2018). In additional analyses, we showed that this item sparks diverse opinions (variation is highest among alternative gender attitude items) and it is highly correlated with these other indicators of support for gendered division of labor, such as “A working mother's relationship with her children can be just as close and warm as that of a non‐working mother”, “The father should earn money, while the mother takes care of the household and the family”, and “A woman is more suited to rearing young children than a man”. Given that this item appears to be highly correlated with other indicators of gender attitudes, we do not have a theoretically based reason to believe that our findings about the impact of the pandemic would be context‐specific. However, this remains an open area for investigation. Next, the analysis is based on a longitudinal sample representative of the Dutch population, but unfortunately the sample size is limited for the specific categories we look at and therefore not large enough to be able to further examine mechanisms in subgroups. A third limitation pertains to the time difference between the baseline measurement in 2017 and the COVID‐19 follow‐up in Spring 2020. This means that we may miss the real size of the COVID‐19 effect if for instance gender attitudes had changed in one direction until just before the onset of COVID‐19 pandemic and then changed course. The finding of no traditional backlash in the investigated gender attitude item for mothers with coresident children could be due to such turnaround if there would have been progression toward more gender progressive attitudes until just before the pandemic in that group. In other words, our finding of “no change” in some cases might in fact be an underestimation of the potential retraditionalizing power of the COVID‐19 pandemic. A further limitation is that our hypotheses are deduced based on the cognitive dissonance theory. However, our analysis is not a strict test of the theoretical mechanism as we do not have information on the emotional experience of the respondent around these changes in the paid and unpaid work routines. We did not measure the discomfort itself as a mediator in the pathway between change in work situation and gender attitude change, which is an avenue for future research.

Despite its limitations, this study on gender attitude change at the onset of the COVID‐19 pandemic forms a useful complement to studies focusing on work–life conflict and couples' division of work and family. It showed that people tend to adjust their gender attitudes to the changes in paid and unpaid work they encountered at the onset of the COVID‐19 pandemic. This might indicate a change that surpasses the specific behavioral response and gives a more broad insight into changing cultural norms around gender and employment.

## Data Availability

The 2017 data used in this study are openly available at http://doi.org/10.4232/1.13560, ZA7500 Data file Version 4.0.0. The 2020 COVID‐19 dataset is available from the corresponding author on reasonable request.

## ENDNOTE

^1^
A number of terms are used to denote gender attitudes: gender ideology, gender role attitude, gender‐related attitudes, and gender egalitarianism. These are the sample sizes before the removal of observations with item nonresponse, and therefore they do not correspond to the sample sizes mentioned in Table 2.

## APPENDIX A Attrition analysis

Table A1 presents the results of an analysis of the attrition between the two waves. We examine the distribution of gender, education level, living situation, age and the gender attitude of interest measured in 2017 for two samples: (1) the 2017 sample and (2) the subsample of those that participated again in 2020. In the ideal scenario without attrition, both samples would be the same; in a scenario of perfectly random drop out, the variable distributions would be exactly the same.

**TABLE A1:** Distribution of 2017 and 2020 sample

Categorical variables	2017 sample				2020 sample			
	Men		Women		Men		Women	
	*N*	%	*N*	%	*N*	%	*N*	%
Gender	590	42.60	795	57.40	367	45.88	433	54.13
Education 2017								
Low	108	19.25	153	19.69	68	19.26	100	23.42
Middle	162	28.88	267	34.36	99	28.05	138	32.32
High	291	51.87	357	45.95	186	52.69	189	44.26
Household type 2017								
Single without children	186	31.53	222	27.92	120	32.70	121	27.94
Living with partner without children, <40	44	7.46	70	8.81	22	5.99	30	6.93
Living with partner without children, ≥40	105	17.80	131	16.48	81	22.07	93	21.48
Living with partner with children	204	34.58	291	36.60	121	32.97	155	35.80
Other	52	8.64	81	10.19	23	6.26	34	7.85

Continuous variables	Mean	SD	Mean	SD	Mean	SD	Mean	SD
Age 2017	44.77	12.86	42.3	13.08	46.87	12.71	44.88	13.09
Family life suffers when woman has full time job 2017	2.14	0.82	2.15	0.88	2.12	0.82	2.17	0.91

*Note*: Data: European Values Study, the Netherlands 2017, 2020. Analytical sample age 18–66. Unweighted data.

The left columns present the unweighted distribution of the 2017 sample, while the columns on the right present the unweighted distribution of the 2020 sample for both men and women. Firstly, we note that attrition is larger for women than for men. The male sample reduces with 37.8% from 590 to 367 respondents, whereas the female sample reduces with 45.5% from 795 to 433 respondents. It is therefore important to examine whether the attrition within the gender groups is related to key variables such as household type, the gender attitude variable of interest as well as socio‐demographic variables such as age, gender and education level. We find that the distribution on the education variable remains rather constant in the 2020 sample, so attrition happened more or less randomly with respect to the educational variable. This is not the same for household type, as we see that those living with a partner and without children aged 40+ are overrepresented and—to a lesser extent ‐ those living with a partner and children are underrepresented in the 2020 sample (meaning they dropped out more). The 2020 samples are also slightly older, meaning that younger people dropped out more. However, and importantly, despite these patterns of attrition, we found that the distribution of our main gender attitude of interest “Family life suffers when a woman has a full‐time job” remained rather constant across the samples.

## APPENDIX B Gender attitudes

**TABLE B1:** Gender attitudes in the Netherlands, 2017

	*N*	Mean	Variance	Coefficient of variation
When a mother works for pay, the children suffer	1756	2.99	0.5051	0.2378
A job is all right but what most women really want is a home and children	1707	2.99	0.5989	0.2584
All in all, family life suffers when the woman has a full‐time job	1741	2.79	0.7101	0.3025
A man's job is to earn money; a woman's job is to look after the home and family	1766	3.38	0.4688	0.2029
On the whole, men make better political leaders than women do	1717	3.22	0.5155	0.2233

*Note:* Data: European Values Studies, 2017, Netherlands. Analytical sample: age 18–67. Weighted data: Agreement with statement. 1 Strongly disagree; 2 Disagree; 3 Agree; 4 Strongly agree.

**TABLE B2:** Correlations between gender role attitude items

	1	2	3	4	5	6
1. A working mother's relationship with her children can be just as close and warm as that of a non‐working mother						
2. A child that is not yet attending school is likely to suffer the consequences if his or her mother has a job	−0.59					
3. Overall, family life suffers the consequences if the mother has a full‐time job	−0.54	0.67				
4. Both father and mother should contribute to the family income	0.24	−0.21	−0.21			
5. The father should earn money, while the mother takes care of the household and the family	−0.43	0.48	0.52	−0.25		
6. Fathers ought to do more in terms of household work than they do at present	0.16	−0.10	−0.12	0.32	−0.18	
7. Fathers ought to do more in terms of childcare than they do at present	0.10	−0.05	−0.08	0.30	−0.14	0.79

*Note*: Data: pre‐Covid wave of LISS (wave 11, collected end of 2018/beginning of 2019), analytical sample: ages 16–67. All listed correlations are significant at *p* < 0.01.

**TABLE B3:** Correlations between gender role attitude items

	1	2	3	4
1. Overall, family life suffers the consequences if the mother has a full‐time job				
2. A woman is more suited to rearing young children than a man	0.43			
3. It is actually less important for a girl than for a boy to get a good education	0.22	0.24		
4. Generally speaking, boys can be reared more liberally than girls	0.27	0.36	0.45	
5. It is unnatural for women in firms to have control over men	0.31	0.33	0.53	0.49

*Note*: Data: pre‐Covid wave of LISS (wave 11, collected end of 2018/beginning of 2019), analytical sample: age 16–67. All listed correlations are significant at *p* < 0.01.
